# Low expression of *NSD1*, *NSD2*, and *NSD3* define a subset of human papillomavirus-positive oral squamous carcinomas with unfavorable prognosis

**DOI:** 10.1186/s13027-021-00347-6

**Published:** 2021-02-15

**Authors:** Steven F. Gameiro, Farhad Ghasemi, Peter Y. F. Zeng, Neil Mundi, Christopher J. Howlett, Paul Plantinga, John W. Barrett, Anthony C. Nichols, Joe S. Mymryk

**Affiliations:** 1 Department of Microbiology and Immunology, The University of Western Ontario, London, ON N6A 3K7 Canada; 2 Department of Surgery, The University of Western Ontario, London, ON N6A 3K7 Canada; 3 Department of Otolaryngology, Head & Neck Surgery, The University of Western Ontario, London, ON N6A 3K7 Canada; 4 Department of Pathology, The University of Western Ontario, London, ON N6A 3K7 Canada; 5 Department of Oncology, The University of Western Ontario, London, ON N6A 3K7 Canada; 6 London Regional Cancer Program, Lawson Health Research Institute, London, ON N6C 2R5 Canada; 7 Department of Otolaryngology - Head and Neck Surgery, Schulich School of Medicine & Dentistry, Western University, Room B3-431A, 800 Commissioners Road East, London, ON N6A 5W9 Canada; 8 London Regional Cancer Program, 790 Commissioners Rd. East, London, Ontario N6A 4L6 Canada

**Keywords:** Head and neck cancer, Head and neck squamous cell carcinoma, HPV, WHSC1, WHSC1L1, Epigenetics, Histone methyltransferase, The Cancer Genome Atlas

## Abstract

**Background:**

Frequent mutations in the nuclear receptor binding SET domain protein 1 (*NSD1*) gene have been observed in head and neck squamous cell carcinomas (HNSCC). *NSD1* encodes a histone 3 lysine-36 methyltransferase. *NSD1* mutations are correlated with improved clinical outcomes and increased sensitivity to platinum-based chemotherapy agents in human papillomavirus-negative (HPV-) tumors, despite weak T-cell infiltration. However, the role of *NSD1* and related family members *NSD2* and *NSD3* in human papillomavirus-positive (HPV+) HNSCC is unclear.

**Methods:**

Using data from over 500 HNSCC patients from The Cancer Genome Atlas (TCGA), we compared the relative level of mRNA expression of *NSD1*, *NSD2*, and *NSD3* in HPV+ and HPV- HNSCC. Correlation analyses were performed between T-cell infiltration and the relative level of expression of *NSD1*, *NSD2*, and *NSD3* mRNA in HPV+ and HPV- HNSCC. In addition, overall survival outcomes were compared for both the HPV+ and HPV- subsets of patients based on stratification by *NSD1*, *NSD2*, and *NSD3* expression levels.

**Results:**

Expression levels of *NSD1*, *NSD2* or *NSD3* were not correlated with altered lymphocyte infiltration in HPV+ HNSCC. More importantly, low expression of *NSD1*, *NSD2*, or *NSD3* correlated with significantly reduced overall patient survival in HPV+, but not HPV- HNSCC.

**Conclusion:**

These results starkly illustrate the contrast in molecular features between HPV+ and HPV- HNSCC tumors and suggest that *NSD1*, *NSD2*, and *NSD3* expression levels should be further investigated as novel clinical metrics for improved prognostication and patient stratification in HPV+ HNSCC.

**Supplementary Information:**

The online version contains supplementary material available at 10.1186/s13027-021-00347-6.

## Introduction

Head and neck squamous cell carcinomas (HNSCC) comprise a group of heterogeneous cancers that arise from multiple anatomical subsites in the head and neck region. Collectively, they represent the 7th most common human cancer type [1] and are often characterized by aggressive local invasion and overall poor prognosis [2]. Historically, tobacco use and alcohol consumption are major risk factors for HNSCC [3]. However, infection with human papillomavirus (HPV) has recently emerged as a major cause of tumors located in the oropharynx. Indeed, HPV-positive (HPV+) HNSCC is increasing at an epidemic rate [4, 5]. Numerous studies have confirmed that HPV-negative (HPV-) and HPV+ HNSCC are molecularly distinct [6]. HPV+ HNSCC constitutively express the viral *E6* and *E7* oncogenes that deregulate cell growth and gene expression [7], at least in part via epigenetic mechanisms [8, 9]. Importantly, clinical outcomes for HPV+ HNSCC are superior to those of HPV- cases [10, 11], allowing for potential stratification of patients into alternative treatment regimens based on HPV status [12].

Histone methylation plays a critical role in the epigenetic control of gene expression. Specialized enzymes methylate/de-methylate individual amino acids that are found on the histone tails H1, H2A, H2B, H3, and H4. The nuclear receptor binding SET domain protein (NSD) family of histone-lysine N-methyltransferases are composed of three paralogous proteins: NSD1, NSD2 (WHSC1), and NSD3 (WHSC1L1). NSD1 mediates the transfer of a methyl group onto H3 lysine-36 (H3K36) and H4 lysine-20 (H4K20) [13]. NSD2 methylates H3 lysine-4 (H3K4) and H4K20, and NSD3 methylates H3K36 [14]. NSD paralogs also methylate non-histone substrates, these include NSD1-mediated methylation of nuclear factor kappa-light-chain-enhancer of activated B cells (NF-κB) [15]; NSD2-mediated methylation of phosphatase and tensin homolog (PTEN) [16]; and NSD3-mediated methylation of interferon regulatory factor 3 (IRF3) and epidermal growth factor receptor (EGFR) [17, 18]. Furthermore, a functional role for NSD2 in the type-I interferon response has also been reported [19]. Moreover, numerous studies have linked the NSD family of methyltransferases with a variety of different cancers [20].

We and others used hierarchical clustering of DNA methylation data to identify a subset of HPV- HNSCC tumors enriched for mutations in the *NSD1* H3K36 methyltransferase [8, 9]. These cluster with tumors expressing wild-type *NSD1* that contain a substitution of K36 to methionine in H3. Thus, mutation of the substrate of NSD1 phenocopies the methylation signature of direct mutational activation of this enzyme. This signature represents approximately 13% of HPV- HNSCC and identifies *NSD1* inactivation as a mechanism of epigenome deregulation [8]. Studies using multiple cohorts have demonstrated that HPV- HNSCC patients with *NSD1* gene alterations exhibited improved survival compared to patients with wild-type *NSD1* tumors [21, 22]. Furthermore, mutation or reduced expression of *NSD1* in HPV- HNSCC has been reported to confer increased sensitivity to platinum-based chemotherapy agents in vitro [22, 23], despite leading to an immunologically “cold” phenotype characterized by slightly reduced T-cell infiltration [24]. Another study suggested that mutation of *NSD1* or *NSD2* leads to significantly better clinical outcomes in HPV- HNSCC of the larynx [25], further supporting an oncogenic role for these methyltransferases in HPV- HNSCC. In contrast, little is known about the role of *NSD1*, or its paralogs *NSD2* and *NSD3* in HPV+ HNSCC, except that mutation of these genes appears to occur at a reduced frequency as compared to HPV- HNSCC [6, 26] and *NSD1* mutation is correlated with reduced survival [22].

In this study, we used data from over 500 HNSCC patients from the Cancer Genome Atlas (TCGA) to compare the expression levels of mRNA for *NSD1*, *NSD2*, and *NSD3* between HPV+ HNSCC, HPV- HNSCC, or normal control tissues. We also investigated the relationship between the levels of expression of *NSD1*, *NSD2,* or *NSD3* with tumor-infiltrating lymphocytes (TILs) in either the HPV+ or HPV- HNSCC samples and whether expression of *NSD1*, *NSD2*, or *NSD3* correlated with overall survival. Our aim was to determine if there were differences in these molecular features that could serve as novel clinical metrics for improved prognostication and patient stratification in HNSCC.

## Material and methods

### Data collection

Patient data from the Cancer Genome Atlas (TCGA), including the Merged Clinical data and Level 3 RNA-Seq by Expectation-Maximization (RSEM) normalized Illumina HiSeq RNA expression data for the HNSCC cohort, was downloaded from the Broad Genome Data Analysis Centers Firehose server (https://gdac.broadinstitute.org/). RNA-seq viral read counts for HPV *E6* and *E7* was extracted from the supplementary data files from Chakravarthy et al. [27]. Patient survival data for the TCGA HNSCC cohort was extracted from the Pan-Cancer Clinical Data Resource [28]. All data utilized in this study can be found in Additional file 1 - Supplementary Table 1 (Table S1).

### RNA expression comparisons

RSEM normalized expression data was extracted and curated as described [29]. Primary patient samples with known HPV status were grouped as HPV+, HPV-, or normal control tissues. This resulted in 73 HPV+, 442 HPV-, and 43 matched normal-adjacent control samples with data available for the HNSCC gene expression analysis. Boxplot comparisons of gene expression was performed using GraphPad Prism v7.0 (Graphpad Software, Inc., San Diego, California, USA) and assembled into final form using Adobe Illustrator (Adobe Systems Inc., San Jose, CA, USA). For the boxplots, center lines show the medians, box limits indicate the 25th and 75th percentiles as determined by Graphpad Prism and whiskers extend 1.5 times the interquartile range from the 25th and 75th percentiles. Statistical significance was calculated using Graphpad Prism v7.0. The statistical *p* values were assigned using a two-tailed non-parametric Mann–Whitney U test.

### Correlation matrix

Level 3 RSEM normalized RNA-seq data for *NSD1*, *NSD2*, and *NSD3* were extracted from the TCGA database and processed into HPV+ and HPV- cohorts as detailed above. As mentioned above, HPV *E6* and *E7* RNA expression data was extracted from Chakravarthy et al. [27]*.* A pairwise Spearman correlation was performed for each of the aforementioned genes. Correlations were performed using RStudio (version 1.2.1335) utilizing the *ggplot2* package [30]. The final correlation matrix figure was assembled using Adobe Illustrator (Adobe Systems Inc., San Jose, CA, USA).

### Survival analyses

RSEM normalized RNA-seq data for each of *NSD1*, *NSD2*, and *NSD3* were converted into a standard score (zscore) and grouped into high, mid, and low expression based on the following criteria: high = z-score > 0.5, mid = − 0.5 < z-score < 0.5, and low = z-score < − 0.5. Five-year overall survival outcomes were compared in both HPV+ and HPV- subsets of patients grouped by either high, mid, or low expression of *NSD1*, *NSD2*, or *NSD3*. Log-rank statistical analysis was performed using GraphPad Prism v7.0 (Graphpad Software, Inc., San Diego, California, USA). Furthermore, log-rank *p* values were assessed for significance after correcting for false discovery rate (FDR) using the Benjamini–Hochberg method with an FDR threshold of 10%. Figures were assembled into final form using Adobe Illustrator (Adobe Systems Inc., San Jose, CA, USA). Univariate analysis was performed through RStudio (version 1.2.1335) based on a Cox Proportional-Hazards Model with the *survival* package (version 2.41–3). Finally, stepwise bidirectional multivariate analysis was performed with the grouped expression of the *NSD* paralogs and the following clinical variables: sex, age, subsite, T stage, N stage, overall stage, HPV type, and smoking history. The smoking history clinical variable for the HPV+ cohort was stratified as heavy smokers (> 20 pack year history) or non-smokers based on our previous study that employed a similar stratification approach [21]. Moreover, patients with no smoking history information, patients with between 1 and 20 pack years, and patients who were listed as current or former smokers but with unknown pack year history were excluded. Statistical *p* values were derived from the Wald test on survival coefficients.

### Correlation analysis for T-cell infiltration status

To estimate T-cell infiltration, we used a previously generated T-cell signature based on mean expression of 13 transcripts [31] as utilized by Brennan et al. [24]. The expression of *NSD1*, *NSD2*, and *NSD3* relative to that of the T-cell infiltration signature were compared in a pairwise fashion and concordance calculated by Spearman’s Rho analysis.

## Results

### Expression of *NSD1*, *NSD2*, and *NSD3* in HNSCC stratified by HPV status

**Fig. 1:**
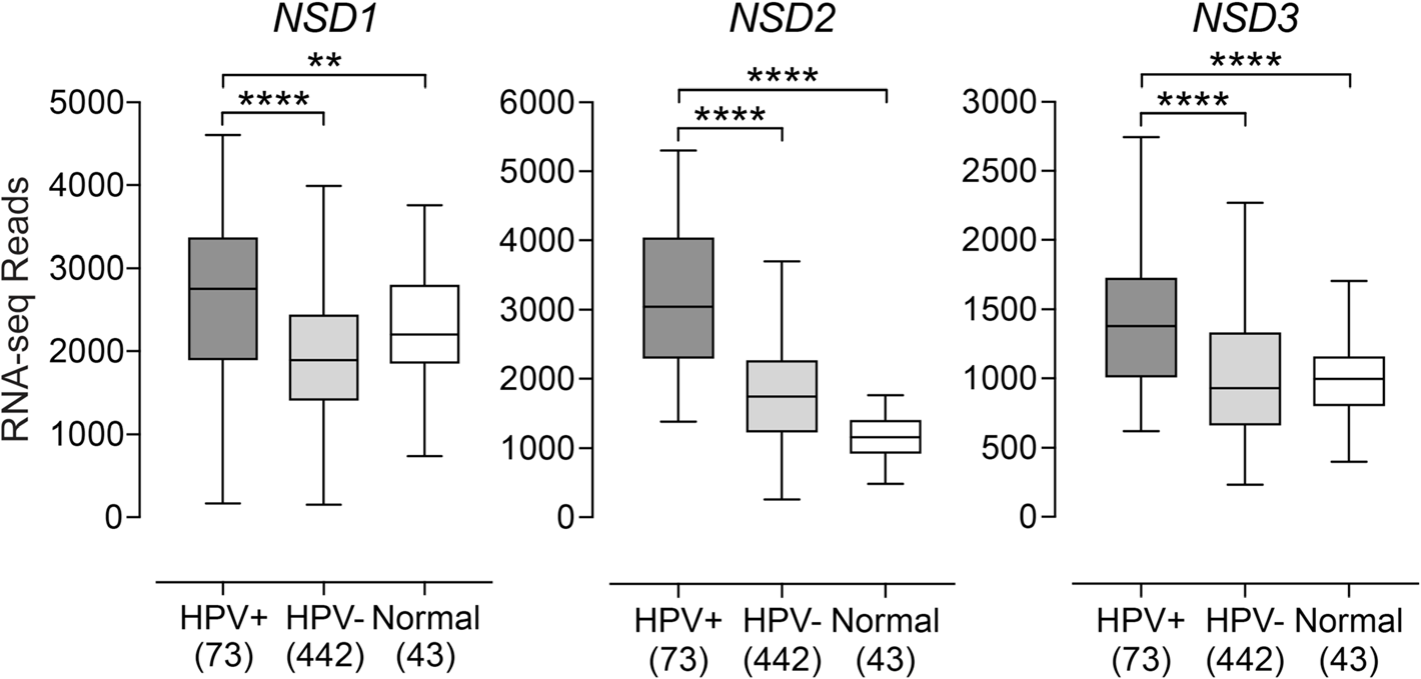
Expression of *NSD1*, *NSD2*, and *NSD3* genes in HNSCC stratified by HPV status. Normalized RNA-seq data was extracted from TCGA database for the HNSCC cohort for HPV+, HPV-, and matched normal-adjacent control tissues. Numbers in brackets refer to the number of samples included in each analysis. Statistical *p* value significance levels are indicated as follows: * *p* ≤ 0.05; ** *p* ≤ 0.01; *** *p* ≤ 0.001; **** *p* ≤ 0.0001; ns – not significant

Given that NSD1 and NSD2 have been linked to various aspects of HPV- HNSCC, including epigenetic alterations, immune status of the tumor, and predicting patient outcome at specific subsites [8, 9, 21–26], we investigated the roles of *NSD1* and its two paralogs *NSD2* and *NSD3* in HPV+ HNSCC. Previous work has suggested that *NSD1* RNA expression can serve as a measure of NSD1 proficiency in HNSCC [24]. As such, we analyzed the TCGA Illumina HiSeq RNA expression data from the HNSCC cohort for expression of all three paralogous genes (Fig. 1). Unexpectedly, the HPV+ samples had significantly increased levels of expression of all three paralogs as compared to HPV- tumors and normal control tissues.

**Fig. 2:**
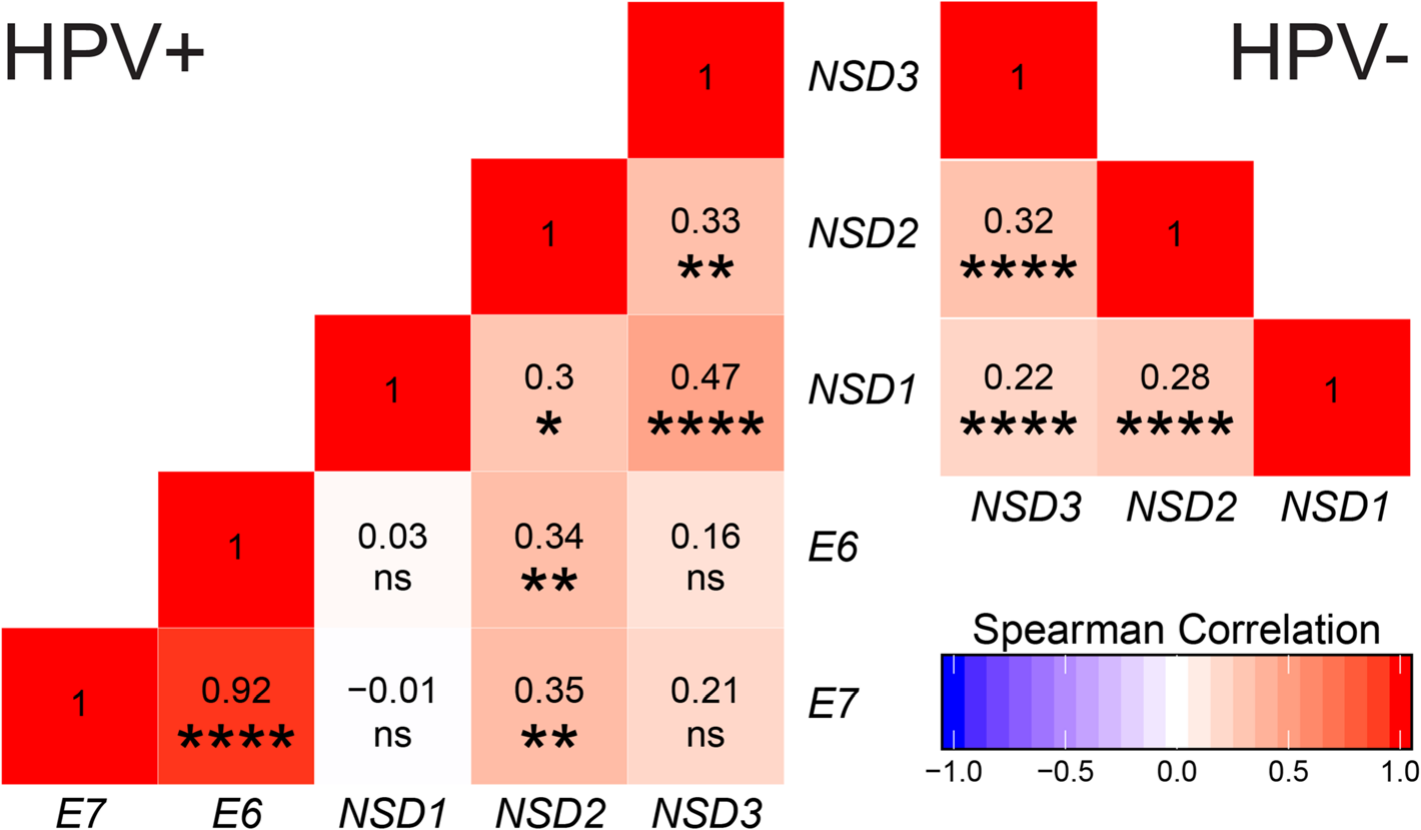
Correlation matrix of *NSD* paralogs and viral oncogene expression. Normalized RNA-seq data for *NSD1*, *NSD2*, *NSD3*, HPV *E6*, and HPV *E7* was compared in a pairwise fashion for both the HPV+ (left) and HPV- (right) TCGA HNSCC cohorts. Numbers indicated in the boxes are the calculated Spearman’s rank correlation coefficient of the indicated gene pairs. Statistical *p* value significance levels are indicated as follows: * *p* ≤ 0.05; ** *p* ≤ 0.01; *** *p* ≤ 0.001; **** *p* ≤ 0.0001; ns – not significant

To determine if this significant increase in expression of *NSD* paralogs in the HPV+ cohort was due to the constitutive expression of viral oncogenes, we correlated HPV *E6* and *E7* mRNA expression levels with those of *NSD1*, *NSD2*, or *NSD3*. Our analysis revealed a statistically significant positive Spearman correlation coefficient between *NSD2* expression and either *E6* or *E7* (Fig. 2). In contrast, the Spearman correlation coefficients between the HPV viral oncogenes and *NSD1* or *NSD3* were not statistically significant (Fig. 2). Thus, the HPV viral oncogenes and *NSD2* have an increasing monotonic relationship that may explain the high expression observed for *NSD2* in the HPV+ HNSCC cohort compared to its HPV- counterpart and normal control tissues. Alternatively, *E6* and *E7* expression could be regulated by *NSD2*.

### HNSCC tumor samples concordantly express *NSD1*, *NSD2*, and *NSD3*

Given that all three *NSD* paralogs are expressed on average at significantly higher levels in HPV+ HNSCC, but only *NSD2* had a statistically significant positive Spearman correlation coefficient with the HPV oncogenes, we next wanted to determine if individual tumors expressed high levels of a single *NSD* paralog on a mutually exclusive basis, or if their expression might be coordinately regulated. We performed pairwise analysis of RNA expression of each paralog with respect to the others for each sample in the HPV+ and HPV- subsets of this cohort (Fig. 2). This pairwise analysis indicated that HPV+ tumors expressing a high level of one paralog express higher relative levels of the other paralogs. Similarly, HPV+ tumors expressing a low level of one paralog express lower relative levels of the other paralogs. A similar correlation was observed for HPV- tumors. Thus, coordinately upregulated expression of all three *NSD* paralogs is frequently observed in HNSCC, regardless of HPV status.

### T-cell infiltration is correlated with *NSD1* expression in HPV-, but not HPV+ HNSCC

**Fig. 3:**
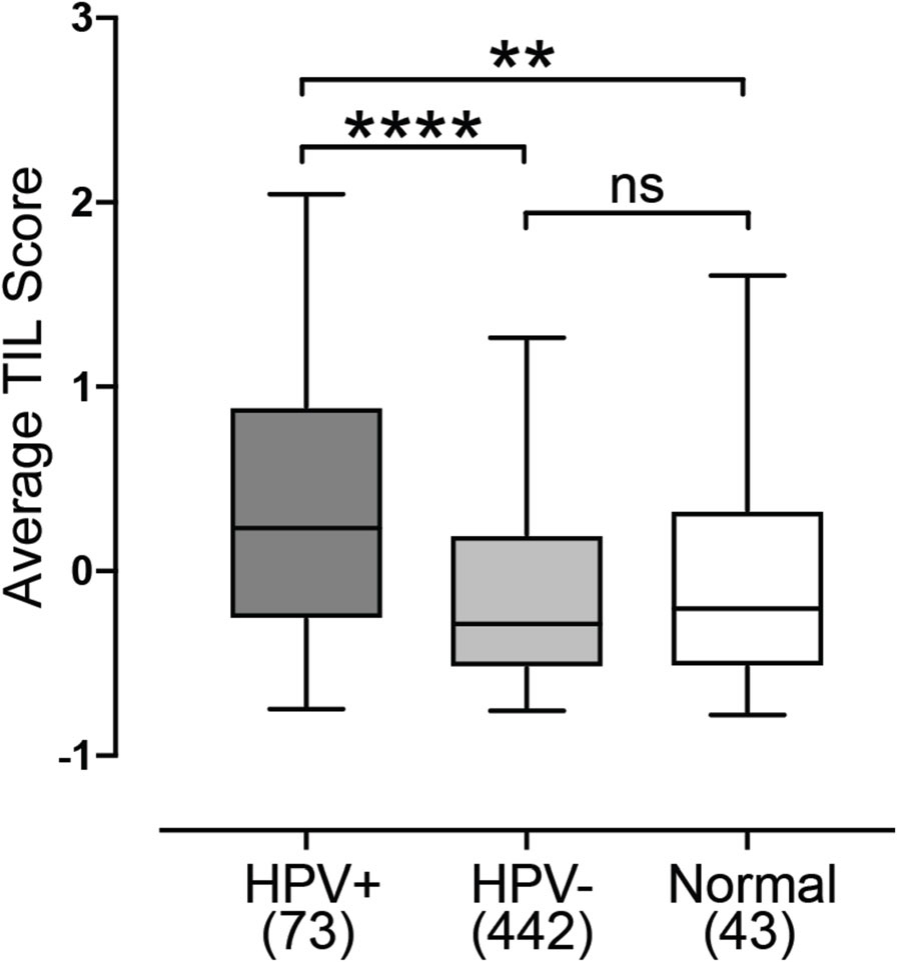
T-cell infiltration is higher in HPV+ HNSCC compared to HPV- and normal control counterparts. A previously reported 13 gene T-cell transcript signature was calculated for HPV+, HPV-, and matched normal-adjacent control tissues. Numbers in brackets refer to the number of samples included in each analysis. Statistical *p* value significance levels are indicated as follows: * *p* ≤ 0.05; ** *p* ≤ 0.01; *** *p* ≤ 0.001; **** *p* ≤ 0.0001; ns—not significant

**Fig. 4:**
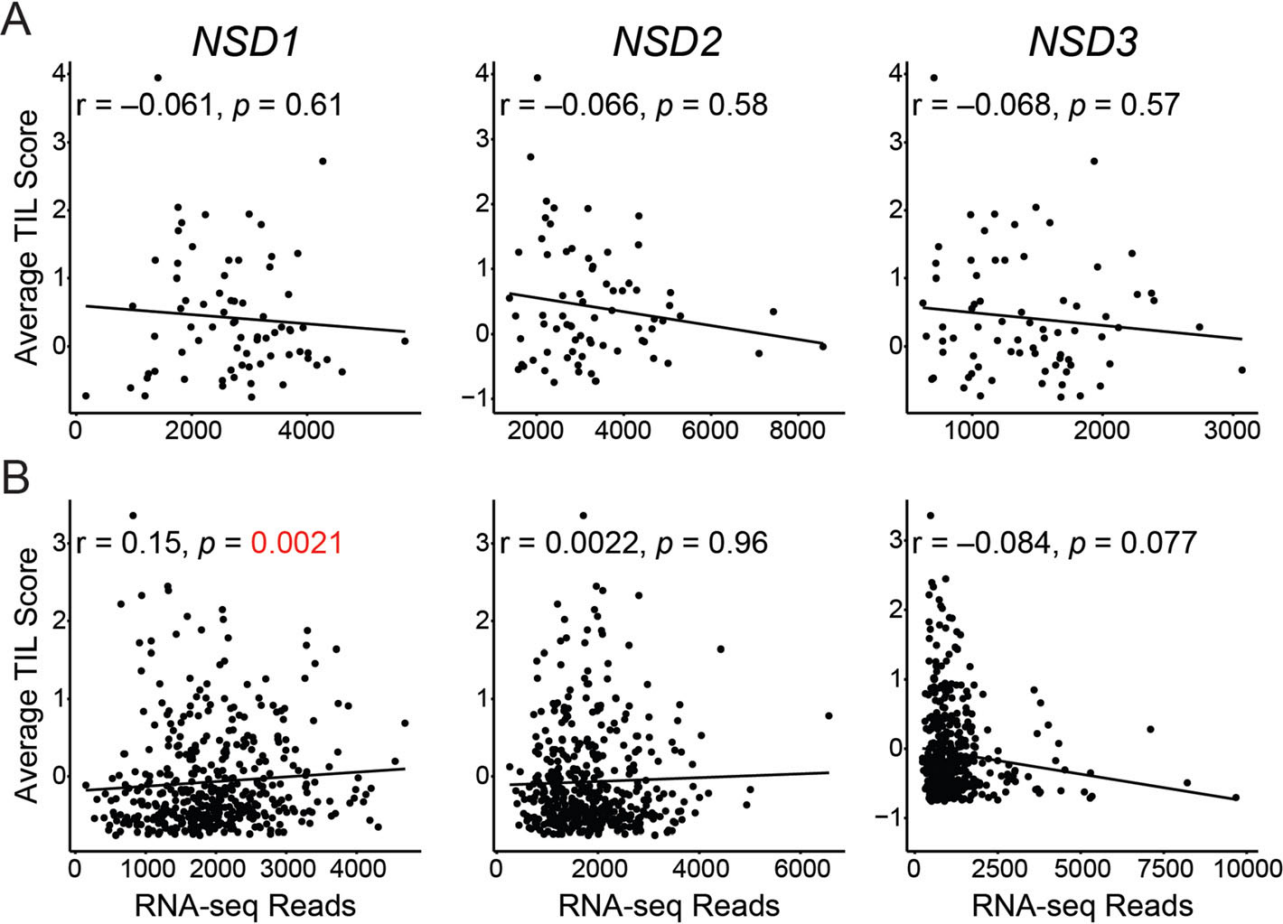
T-cell infiltration is not correlated with *NSD1* expression in HPV+ HNSCC, but is weakly correlated in HPV- HNSCC. A previously reported 13 gene T-cell transcript signature was calculated for each sample in the TCGA HNSCC cohort and compared pairwise with normalized RNA-seq data for *NSD1*, *NSD2*, and *NSD3* for HPV+ (**a**) and HPV- (**b**) HNSCC samples. For each *NSD* paralog, the correlation with the T-cell transcript signature was calculated by Spearman’s Rho analysis

Despite improved prognosis, *NSD1* mutation in HPV- HNSCC has been associated with an immunologically “cold” tumor microenvironment associated with slightly low levels of TILs [24]. Furthermore, weak T-cell infiltration was also associated with decreased *NSD1* expression in other patient-derived datasets for HPV- tumors and experimental tumor models with HPV- HNSCC cells [24]. Utilizing the same 13 gene T-cell signature defined by Spranger et al. [31], T-cell infiltration was clearly higher in HPV+ HNSCC compared to the HPV- counterparts and normal control tissues (Fig. 3), consistent with the immunologically “hot” phenotype associated with HPV+ HNSCC [32, 33]. We next determined whether *NSD1* levels were inversely associated with T-cell infiltration in HNSCC as reported previously for HPV- HNSCC [24]. We found no significant correlation between markers of T-cell infiltration and expression of either *NSD1*, *NSD2*, or *NSD3* in the HPV+ HNSCC cohort (Fig. 4a). In contrast, a weak but statistically significant positive Spearman correlation coefficient with *NSD1* expression was observed for HPV- HNSCC (Fig. 4b), which is in agreement with the aforementioned study by Brennan et al. [24]. However, no statistically significant Spearman correlation coefficients were observed between either *NSD2* or *NSD3* expression and TIL score in the HPV- HNSCC cohort (Fig. 4b). Thus, a possible connection between *NSD1* expression and T-cell infiltration into the tumor microenvironment appears unique to HPV- HNSCC samples and is not shared with the other *NSD* paralogs.

### Low expression levels of either *NSD1*, *NSD2*, or *NSD3* predict reduced overall survival in HPV+ HNSCC

**Fig. 5:**
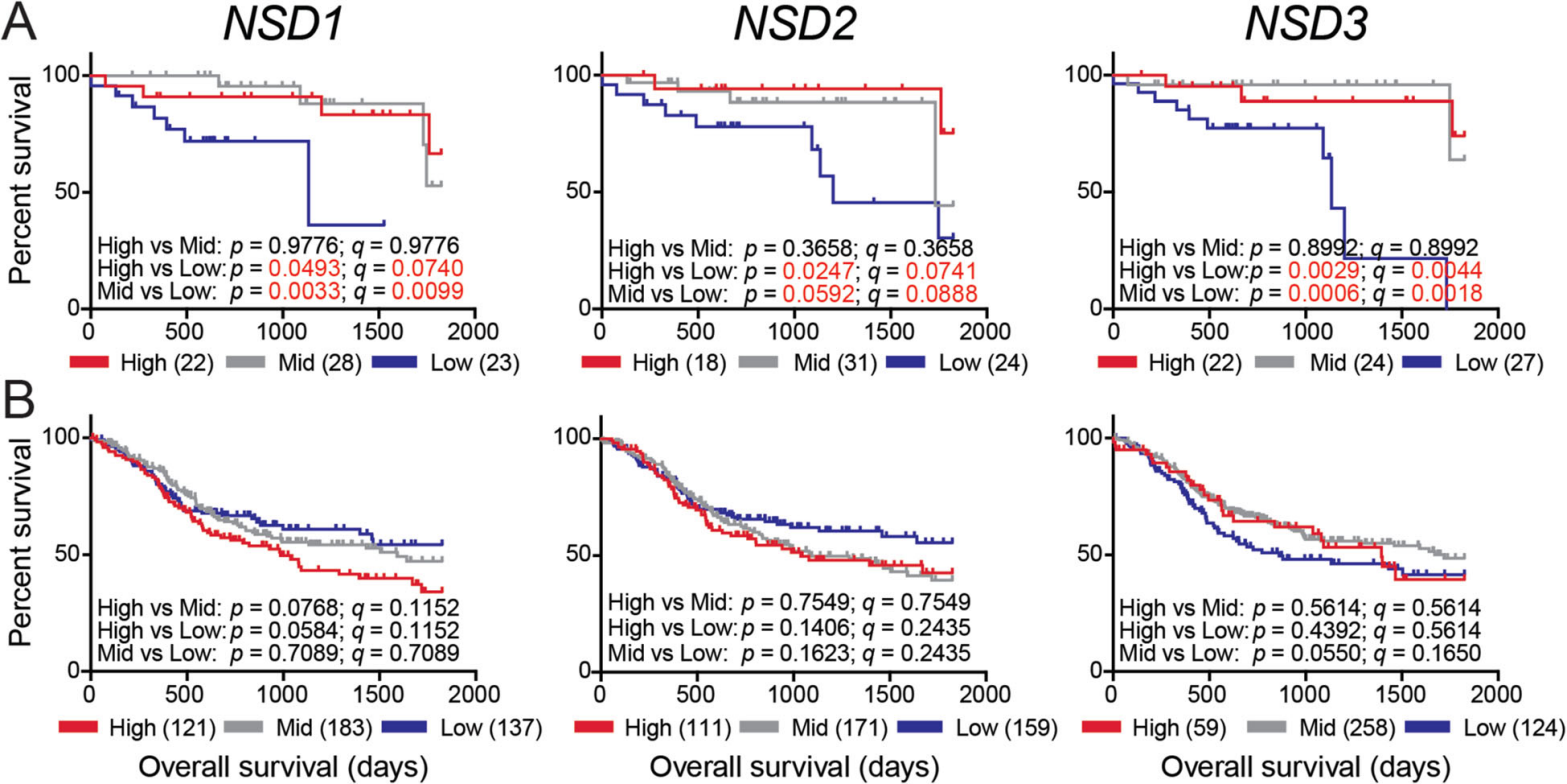
Low *NSD1*, *NSD2*, or *NSD3* expression is strongly associated with reduced survival in patients with HPV+ but not HPV- HNSCC. Overall survival of patients grouped by high, mid, or low expression of *NSD1*, *NSD2*, or *NSD3* in the HPV+ (**a**) and HPV- (**b**) HNSCC cohorts. Comparison between groups were calculated with the 2-sided log-rank test (*p* value) and multiple comparisons corrected with the Benjamini–Hochberg method utilizing an FDR threshold of 10% (*q* value). Numbers in brackets indicate the number of samples included in each expression group

Our previous study did not observe any significant relationship between the NSD1/H3K36M methylation signature and clinical outcomes in the TCGA HNSCC cohort [8]. More recently, the presence of damaging mutations in *NSD1* or *NSD2* have been linked to significantly improved patient survival, but only in HPV- tumors [21, 22, 25]. We subdivided the HPV+ and HPV- HNSCC dataset based on high (z-score > 0.5), mid (− 0.5 < z-score < 0.5), or low (z-score < − 0.5) *NSD1*, *NSD2*, or *NSD3* RNA expression and calculated the impact of expression on overall patient survival (Fig. 5). Unexpectedly, low expression of either *NSD1*, *NSD2*, or *NSD3* predicted markedly reduced survival in HPV+ HNSCC over those patients with tumors expressing the *NSD* paralogs in the mid and high expression groups. Furthermore, this sharp overall decrease in clinical outcome was also statistically significant (Fig. 5a). In contrast, there was no statistically significant impact of either *NSD1*, *NSD2*, or *NSD3* expression on overall survival for patients with HPV- HNSCC (Fig. 5b).

To determine the extent that each of the NSD family members could influence patient outcomes in the HPV+ HNSCC cohort, we generated a hazard ratio (HR) for each gene by univariate analysis (Table 1). As expected, the HRs for either *NSD1* (low vs high expression and low vs mid expression), *NSD2* (low vs high expression), and *NSD3* (low vs high expression and low vs mid expression) were statistically significant, with *NSD2* (low vs mid expression) trending towards significance. Since expression of these genes might not be independent predictors of survival, we analyzed the relationship between survival and our gene expression groups (high, mid, or low expression) for *NSD1*, *NSD2*, and *NSD3* concurrently by multivariate analysis (Table 1). In addition to the expression of the *NSD* paralogs, we also included clinical variables that could influence outcome in our multivariate analysis, such as: sex, anatomical subsite, T stage, N stage, overall stage, HPV type, and smoking history (Table 1). The results of our multivariate analysis revealed that the HRs for *NSD1* (low vs high expression) and *NSD3* (low vs high expression and low vs mid expression) remained significant (HR = 32.88, 95% confidence interval (CI) = 3.23–333.33, *p* = 0.003; HR = 103.1, 95% CI = 8.55–1.24e03, *p* = 0.0003; HR = 135.3, 95% CI = 6.62–2.76e03, *p* = 0.001, respectively), indicating that low expression of *NSD1* and *NSD3* is a significant, and potentially independent, contributor to overall survival in the HPV+ HNSCC cohort. The final multivariate model also included age (HR = 0.13, 95% CI = 0.023–0.70, *p* = 0.02), overall stage (HR = 0.034, 95% CI = 0.004–0.33, *p* = 0.003), and HPV type (HR = 13.03, 95% CI = 2.46–69.13, *p* = 0.003) as statistically significant contributing factors to survival.

**Table 1:** Univariate and multivariate analysis of *NSD* paralog expression and clinical variables and their association with overall survival in the HPV+ HNSCC cohort

		Univariate Analysis		Multivariate Analysis	
**Variables**		**HR (95% CI)**	***P*** **value**	**HR (95% CI)**	***P*** **value**
Sex	Male vs Female	0.81 (0.18–3.62)	0.78		
Age	< 60 vs ≥ 60	0.96 (0.32–2.83)	0.94	0.13 (0.023–0.70)	*0.02*
Subsite	Oral Cavity vs Oropharynx	2.82 (1.02–7.80)	*0.045*	2.51 (0.55–11.48)	0.23
	Other vs Oropharynx	1.54e-08 (0 - Inf)	1.00	3.72e-10 (0 - Inf)	1.00
	Oral Cavity vs Other	1.84e08 (0 – Inf)	1.00	6.75e09 (0 - Inf)	1.00
T Stage	T3 - T4 vs T1 - T2	1.03 (0.36–2.91)	0.96		
N Stage	N2b - N3 vs N0 - N2a	0.41 (0.14–1.19)	0.10	0.19 (0.034–1.047)	0.06
Overall Stage	IV vs I - III	0.76 (0.26–2.24)	0.62	0.034 (0.004–0.33)	*0.003*
HPV Type	Other vs 16	3.33 (1.14–9.78)	*0.028*	13.03 (2.46–69.13)	*0.003*
Smoking History	Heavy Smoker vs Non Smoker	1.57 (0.47–5.22)	0.47		
*NSD1*	Low vs High Expression	6.15 (1.43–26.32)	*0.015*	32.88 (3.23–333.33)	*0.003*
	Low vs Mid Expression	5.95 (1.49–23.81)	*0.012*	4.94 (0.84–29.41)	0.08
	High vs Mid Expression	0.97 (0.24–3.91)	0.96	0.15 (0.018–1.23)	0.08
*NSD2*	Low vs High Expression	5.08 (1.08–23.88)	*0.04*		
	Low vs Mid Expression	2.93 (0.89–9.67)	0.08		
	High vs Mid Expression	0.58 (0.10–3.29)	0.54		
*NSD3*	Low vs High Expression	10.03 (1.98–50.94)	*0.005*	103.1 (8.55–1.24e03)	*0.0003*
	Low vs Mid Expression	10.63 (2.03–55.62)	*0.005*	135.3 (6.62–2.76e03)	*0.001*
	High vs Mid Expression	1.06 (0.17–6.63)	0.95	1.31 (0.12–14.34)	0.82

*P*<0.05 are in italic

## Discussion

In general, the clinical management of HNSCC is complex, often associated with significant treatment induced morbidities and associated with unacceptably low clinical outcomes. HPV-dependent tumors of the oropharynx are a notable exception, as this subset of HNSCC exhibits dramatically better clinical outcomes. As a result, there is an effort to de-intensify treatment for patients with HPV+ disease in an effort to reduce the acute and chronic toxicities associated with the aggressive treatment protocols necessary for treatment of HPV- HNSCC [12]. Nevertheless, approximately 15–20% of patients with HPV+ disease still fail treatment. The cause of their treatment failure is still unknown, although there is some evidence that outcome depends on HPV genotype and clinical parameters such as tumor size and patient smoking history [10, 34, 35]. However, additional prognostic biomarkers that accurately predict the level of therapeutic intensity necessary for effective treatment are urgently needed.

A variety of evidence indicates that the *NSD1* methyltransferase gene and its paralogs are actively involved in the development of a subset of HNSCC [8] and that their mutation status can predict clinical outcome in HPV- HNSCC [21, 25]. Using TCGA data, we found that HPV+ tumors expressed statistically significant higher levels of *NSD1*, *NSD2*, and *NSD3* compared to their HPV- counterparts and normal control tissues (Fig. 1). The unexpected observation that all three paralogs were upregulated in the HPV+ cohort could be related to the constitutive expression of the viral *E6* and *E7* oncogenes. Correlation of the expression of *E6* and *E7* mRNA with those of *NSD1, NSD2,* or *NSD3* in a pairwise fashion indicated that only *NSD2* had a statistically significant correlation with either *E6* or *E7* (Fig. 2). We also investigated if individual tumor samples expressed high levels of a single *NSD* paralog on a mutually exclusive basis, or if their expression might be coordinately regulated. Our pairwise analysis indicated that tumors expressing high levels of one of these methyltransferases expressed high levels of the other two paralogs (Fig. 2). This coordinate expression pattern was observed in both the HPV+ and HPV- HNSCC cohorts. Thus, coordinate upregulation of the *NSD* paralogs does not appear to be a specific consequence of HPV oncogene expression, as it is observed in both HPV+ and HPV- tumors.

One study reported that mutations in *NSD1*, or low *NSD1* expression leads to an immune “cold” phenotype in HPV- HNSCC [24]. This was characterized using a 13 gene TIL signature developed from melanoma studies [31]. As HPV+ HNSCC are generally considered immune “hot” tumors, with higher immune infiltration and CD8+ T-cell activation compared to HPV- HNSCC [32], we tested whether expression levels of the *NSD* paralogs was also linked to TIL levels in HPV+ samples. Despite confirming a weak correlation between *NSD1* RNA expression and TIL signature in HPV- HNSCC (Fig. 4b), and confirming that the TIL signature is generally higher in HPV+ HNSCC compared to both HPV- HNSCC and normal control tissues (Fig. 3), no significant correlation was observed for *NSD1*, *NSD2*, or *NSD3* in HPV+ HNSCC (Fig. 4a). Thus, expression of the *NSD* paralogs is not correlated with the infiltrating T-cell component of the tumor microenvironment in HPV+ HNSCC. This illustrates yet another difference between HPV+ and HPV- HNSCC. Interestingly, no significant relationship between *NSD2* or *NSD3* expression with the TIL signature was detected in the HPV- HNSCC cohort. This was unexpected, given the positive correlation with *NSD1*, and the observation that *NSD* paralogs appear to be expressed in a coordinate fashion regardless of HPV status (Fig. 2).

Damaging mutations in *NSD1* and *NSD2* were reported to define a subset of stage 3 and 4 laryngeal tumors with favorable prognosis [25]. In addition, mutations in *NSD1* are also present more frequently in heavy smokers and correlated with improved overall survival [21]. As mutations of *NSD* paralogs other than *NSD1* are relatively infrequent in the HPV+ TCGA HNSCC cohort [6], we instead chose to assess if *NSD1*, *NSD2*, or *NSD3* RNA expression was related to overall survival (Fig. 5). A previous report established that *NSD1* transcript expression levels are a reasonable estimate of NSD1 proficiency [32]. Therefore, we grouped the HPV+ and HPV- HNSCC cohorts based on high (z-score > 0.5), mid (− 0.5 < z-score < 0.5), or low (z-score < − 0.5) expression of each paralog and determined overall survival. Importantly, a dramatic and significant decrease in survival is clearly correlated with reduced *NSD1*, *NSD2*, or *NSD3* expression in HPV+ samples (Fig. 5a). These survival differences in the *NSD1* and *NSD3* analyses were independent predictors of survival based on multivariate analysis, with very large effects on relative risk of death (Table 1). It is important to stress that for HPV+ samples, low levels of *NSD* expression predict poor outcome. In stark contrast, damaging mutations in *NSD1* and *NSD2* predict a favourable prognosis in HPV- HNSCC. Thus, NSDs could potentially serve as prognostic tools in completely different ways in different HNSCC types. Importantly, despite the obvious potential for using *NSD* gene expression levels as a tool to identify those HPV+ patients with poor prognosis, *NSD* levels could also be used as a guide to select those patients that could benefit from treatment de-intensification. For example, those patients in the upper two-thirds of *NSD* expression exhibit excellent outcomes that may allow less aggressive treatment, while those in the lower third exhibit poor outcomes that may warrant more aggressive treatment.

Given that the *NSD* paralogs all function as H3K36 methyltransferases, it would seem likely that those HPV+ tumors expressing high NSD levels might exhibit methylation and/or gene expression profiles that differ from those expressing low *NSD* levels. However, intensive efforts based on hierarchical clustering did not identify HPV+ subsets with different methylomes that predict clinical outcomes [8]. Intriguingly, NSDs also methylate and regulate the activities of other non-histone substrates, including the proinflammatory NF-κB transcription factor [15, 36] and IRF3, a key activator of type-I interferon transcription [17]. As both of these transcription factors play key roles in antiviral immunity, reduced expression in HPV+ HNSCC might limit innate immune responses that help clear these virally induced tumors. This is further supported by the observation that *NSD2* knockout impaired the ability of type-I interferon to induce expression of an antiviral gene response [19]. However, *NSD* levels in HPV+ HNSCC are not correlated with an increased TIL score, suggesting that any potential effects on innate immunity do not correlate with increased infiltration of these already highly infiltrated tumors.

## Conclusions

Taken together, this study provides strong evidence that the NSD methyltransferases play opposite roles within HPV+ and HPV- HNSCC. Most importantly and paradoxically, while mutation/loss of function of *NSD* members predicts improved clinical outcome in HPV- HNSCC, low level expression of these genes is a very strong predictor of poor outcome in HPV+ HNSCC. Thus, further investigation of the expression levels of *NSD1*, *NSD2*, and *NSD3* is warranted, as these could serve as rapidly exploitable prognostic biomarkers in HPV+ HNSCC, providing a metric for appropriate treatment de-intensification.

## Supplementary Information

**Additional file 1.**

## Data Availability

The following are available online: Additional file 1- **Table S1** HNSCC clinical and molecular characteristics.

## Abbreviations

EGFR: Epidermal growth factor receptor
FDR: False discovery rate
H3K4: H3 lysine-4
H4K20: H4 lysine-20
H3K36: H3 lysine-36
HR: Hazard ratio
HNSCC: Head and neck squamous cell carcinomas
HPV: Human papillomavirus
HPV-: HPV-negative
HPV+: HPV-positive
IRF3: Interferon regulatory factor 3
NF-κB: Nuclear factor kappa-light-chain-enhancer of activated B cells
NSD: Nuclear receptor binding SET domain protein
PTEN: Phosphatase and tensin homolog
RSEM: RNA-Seq by Expectation-Maximization
TCGA: The Cancer Genome Atlas
TILs: Tumor-infiltrating lymphocytes
